# A new nomogram to predict in-hospital mortality in patients with acute decompensated chronic heart failure and diabetes after 48 Hours of Intensive Care Unit

**DOI:** 10.1186/s12872-024-03848-5

**Published:** 2024-04-06

**Authors:** Linlin Liu, Lei Feng, Cheng Lu, Jiehan Zhang, Ya Zhao, Lin Che

**Affiliations:** 1 Department of Cardiology, Seventh People’s Hospital of Shanghai University of Traditional Chinese Medicine, Shanghai, 200137 China; 2 Department of Cardiology, Shanghai Tongji Hospital, Tongji University School of Medicine, No. 389, Xincun Rd, Putuo District, Shanghai, 200065 China; 3 Department of Cardiology, Kong Jiang Hospital Of Yangpu District, Shanghai, 200093 China

**Keywords:** Acute decompensated chronic heart failure, Diabetes, Mortality, Nomogram, Prediction model

## Abstract

**Background:**

The study set out to develop an accurate and clinically valuable prognostic nomogram to assess the risk of in-hospital death in patients with acute decompensated chronic heart failure (ADCHF) and diabetes.

**Methods:**

We extracted clinical data of patients diagnosed with ADCHF and diabetes from the Medical Information Mart for Intensive Care III database. Risk variables were selected utilizing least absolute shrinkage and selection operator regression analysis, and were included in multivariate logistic regression and presented in nomogram. bootstrap was used for internal validation. The discriminative power and predictive accuracy of the nomogram were estimated using the area under the receiver operating characteristic curve (AUC), calibration curve and decision curve analysis (DCA).

**Results:**

Among 867 patients with ADCHF and diabetes, In-hospital death occurred in 81 (9.3%) patients. Age, heart rate, systolic blood pressure, red blood cell distribution width, shock, β-blockers, angiotensin converting enzyme inhibitors or angiotensin receptor blockers, assisted ventilation, and blood urea nitrogen were brought into the nomogram model. The calibration curves suggested that the nomogram was well calibrated. The AUC of the nomogram was 0.873 (95% CI: 0.834–0.911), which was higher that of the Simplified Acute Physiology Score II [0.761 (95% CI: 0.711–0.810)] and sequential organ failure assessment score [0.699 (95% CI: 0.642–0.756)], and Guidelines-Heart Failure score [0.782 (95% CI: 0.731–0.835)], indicating that the nomogram had better ability to predict in-hospital mortality. In addition, the internally validated C-index was 0.857 (95% CI: 0.825–0.891), which again verified the validity of this model.

**Conclusions:**

This study constructed a simple and accurate nomogram for predicting in-hospital mortality in patients with ADCHF and diabetes, especially in those who admitted to the intensive care unit for more than 48 hours, which contributed clinicians to assess the risk and individualize the treatment of patients, thereby reducing in-hospital mortality.

**Supplementary Information:**

The online version contains supplementary material available at 10.1186/s12872-024-03848-5.

## Introduction

Heart failure (HF) is a clinical syndrome with significant abnormalities in cardiac function and structure due to multiple etiologies, and its high morbidity, multiple hospital admissions, and high mortality rates threaten the quality of life and cause a huge socioeconomic burden. In the United States, the prevalence of HF in adults is 1–2%, and the 1-year all-cause mortality rate is 17% [1]. In China, the number of HF patients reached 8.9 million, and the mortality rate of hospitalized patients reached 2.8%, with an increasing trend [2, 3]. In addition, diabetes is a very universal comorbidity in HF, with approximately 20–40% of patients having diabetes in combination, and the risk of adverse events (repeated admission rate, hospital mortality) is markedly higher in HF patients with diabetes in combination compared to those without diabetes [4–6].

The prognosis of patients with acute decompensated chronic heart failure (ADCHF) and diabetes is influenced by several factors, such as age, race, comorbidities, electrolyte disturbances, treatment strategies, etc. Different studies have reported in-hospital mortality ranging from 20 to 60% in acute heart failure (AHF) [7–9]. Timely and accurate prediction of the risk of in-hospital mortality for such patients based on their clinical information can assist clinicians in rapidly screening high-risk patients and implementing individualized treatment strategies in advance, which will be an effective way to improve in-hospital clinical prognosis. In recent years, nomogram has been used as a visualization tool that can predict disease prognosis [10]. Although studies have been conducted using it as an ideal model to predict poor prognosis in HF, the models developed have poor predictive performance or inconsistent results and do not give a definitive conclusion in clinical practice. More importantly, studies on death during hospital in patients with ADCHF and diabetes are rare, and a simple and well discriminating model to predict the hospital mortality in this group of patients is lacking.

Therefore, this study was conducted to develop and validate a nomogram model to predict hospital mortality based on clinical and laboratory indicators of patients diagnosed with ADCHF and diabetes in the Medical Information Mart for Intensive Care III (MIMIC- III) database, which would help clinicians in risk stratification of patients and guide decision making.

## Methods

### Database and study population

Data for the retrospective study were collected based on the publicly available MIMIC-III database [11], which contains de-identification-related clinical data (e.g., demographics, comorbidities, drug use, laboratory indicators, etc.) of patients admitted to the intensive care unit (ICU) of Beth Israel Deaconess Medical Center from 2001 to 2012. Notably, the institutional review boards of the Massachusetts Institute of Technology (Cambridge, Massachusetts, USA) and Beth Israel Deaconess Medical Center (Boston, Massachusetts, USA) have approved the free access of researchers around the world to the MIMIC-III database for various studies. In addition, since this study is a retrospective study, written informed consent is not required.

**Fig. 1:**
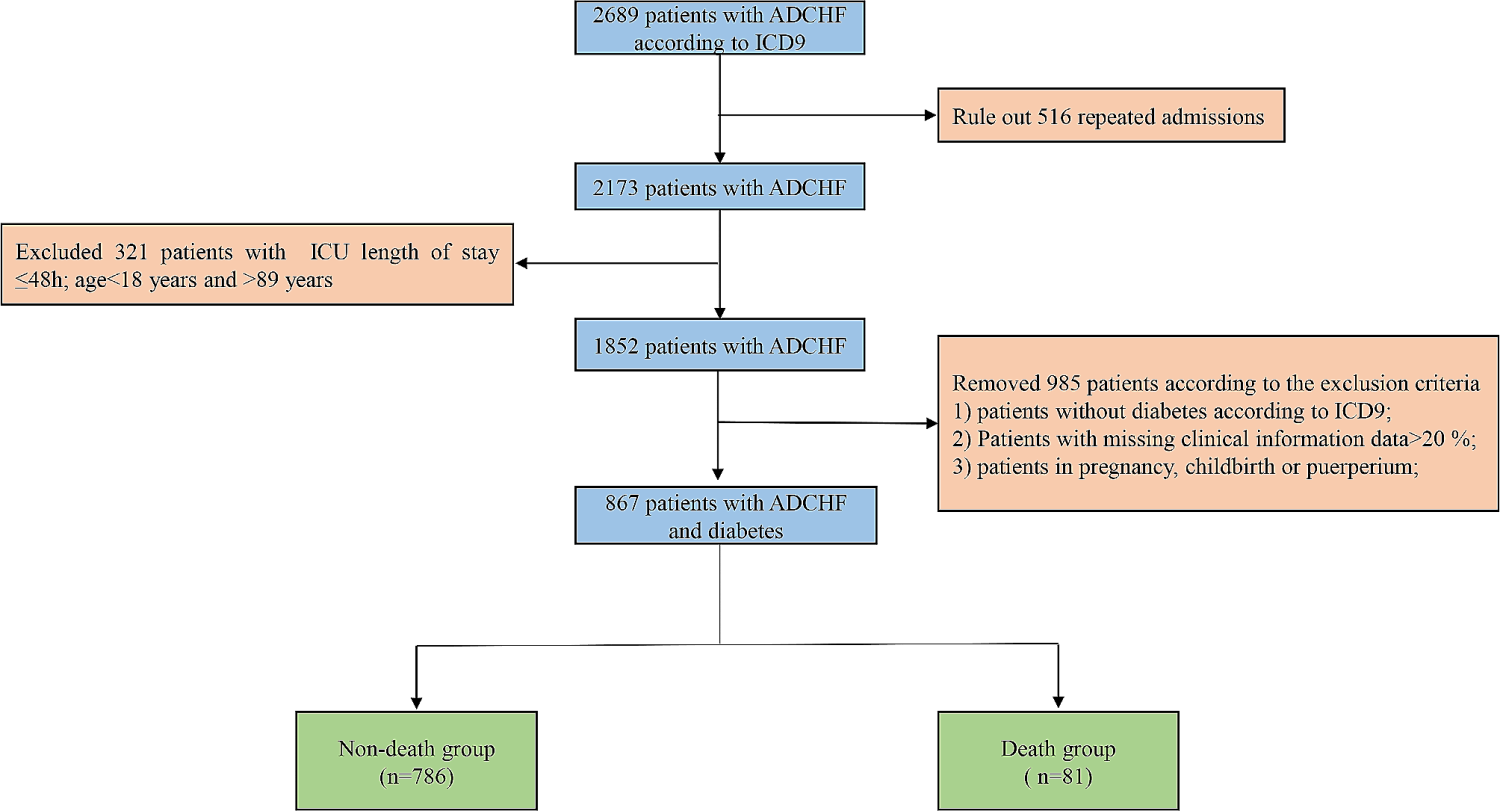
Flow chart of inclusion and exclusion process of patients admitted with ADCHF and diabetes ADCHF: Acute Decompensated Chronic Heart Failure; ICD: International Classification of Diseases; ICU: Intensive Care Unit

This study included patients admitted for diagnosis of ADCHF and diabetes by using International Classification of Diseases (ICD)-9 diagnosis codes between 2001 and 2012. Inclusion criteria: (1) Adults under 89 years old; (2) ICU stay longer than 48 h; (3) Repeatedly hospitalized patients, only retain the first admission to ICU patients, the specific process was shown in Fig. 1. Patients with clinical information data missing > 20% were excluded from this study. We also counted details of all missing values (Supplementary Table 1).

Besides, hospital mortality, as the main endpoint of this study, was defined as all deaths caused by any cause during hospitalization.

### Candidate predictors

We extracted the demographic and health-related data of the study participants from the database. Demographics included age, gender, height, weight, obesity, and smoking status; vital signs contained heart rate (HR), systolic blood pressure (SBP), diastolic blood pressure (DBP), and oxygen saturation. Laboratory test indicators included white blood cell, hemoglobin, platelet, red blood cell distribution width (RDW), serum creatinine (Scr), blood urea nitrogen (BUN), glucose, anion gap (GA), chloride, serum potassium, serum sodium, bicarbonate, partial thromboplastin time (PTT), troponin, N-terminal pro brain natriuretic peptide (NT- proBNP), Glycosylated hemoglobin A1c (HbA1c). Comorbidities included acute coronary syndrome (ACS), hypertension (HP), hyperlipidemia, chronic obstructive pulmonary disease (COPD), cerebral infarction, cardiogenic shock, ventricular arrhythmias, history of prior myocardial infarction (prior-MI), chronic renal dysfunction (CKD), atrial fibrillation (AF), and acute renal impairment (AKI). Medications used during hospitalization include angiotensin-converting enzyme inhibitors (ACEI) or angiotensin receptor blockers (ARBs), β-blockers, aspirin, anticoagulant drugs, diuretics, digoxin, insulin; adjuvant therapy includes continuous renal replacement therapy (CRRT), assisted ventilation. Common scoring systems used in the ICU included the Simplified Acute Physiology Score II (SAPS II) and the Sequential Organ Failure Assessment (SOFA) score. It was important to note that all laboratory indicators were obtained from data first tested within 24 h of admission.

### Statistical analysis

Stata software (V.11.2) and R (V.3.6.2, Vienna, Austria) software were adopted for data processing, statistical analysis, and plotting. All participants were first divided into No-death and death groups based on their survival status at discharge, and baseline characteristics of the two groups were compared. Continuous variables were expressed as mean ± standard deviation or median (interquartile distance, IQR) and compared using student t test or Mann-Whitney U test, categorical variables were expressed as frequency (percentage) and compared using χ2 or Fisher’s exact test. During data processing, the Winsorize function was used to mitigate the effects of outliers. When the continuous variables in the population were normally distributed, the missing value was replaced by the mean, otherwise, the missing value was replaced by the median. The least absolute shrinkage and selection operator (LASSO) regression and logistics multivariate analysis were adopted to select the important risk variables for predicting hospital mortality. The final prediction model results were visualized using a nomogram. The area under the receiver operating characteristic curve (AUC), known as the consistency index (C-index), calibration curves, and Hosmer-Lemeshow (H-L) test were used to evaluate the goodness-of-fit and calibration performance of the nomogram. Decision curve analysis (DCA) was adopted to determine the nomogram ‘s net benefit. Bootstrap methods (1000 resampling) were used to obtain corrected C-indexes to assess the internal validation of the model performance. When *P*-values was less than 0.05, indicating significant differences.

## Results

### Clinical baseline characteristics of the study population with ADCHF and diabetes

After rigorous screening, 867 patients who met the criteria were put into the final analysis. of them, the median age was 72.0 (63.7–80.1) years, 64.2% were male, and 81 (9.3%) died in hospital. All patients were grouped into Non-death and Death groups based on hospital survival, and the clinical baseline characteristics of the two groups are shown in Table 1. Variables such as age, HR, SBP, DBP, Scr, RDW, AG, chloride, serum sodium, and BUN were statistically different in the two groups. In addition, compared with the Non-death group, patients in the Death group had more comorbidities such as shock, AF, and AKI (all *P* < 0.001); In terms of treatment strategies, patients in the Death group used CRRT and assisted respiratory therapy more frequently (*P* < 0.05), while medications such as ACEI/ARBs, anticoagulant drugs, statins, insulin therapy, and β-blockers were less frequent (*P* < 0.05). Not surprisingly, the hospitalization time as well as the length of ICU stay were significantly longer in the Death group, and the SOFA score and SAPII were remarkably higher (all *P* < 0.01).

**Table 1:** Baseline characteristics of Non-death and Death groups

Variables	Total (*n* = 867)	Non-death (*n* = 786)	Death	*P*
			(*n* = 81)	
Age, years	72.0(63.7–80.1)	71.6(63.0-79.8)	75.1(68.7–82.8)	0.001^**^
Gender, male	458(52.8)	414(52.7)	44(54.3)	0.777
Ethnicity, black	136(15.7%)	130(16.5%)	6(7.4%)	0.036*
Smoke	231(26.6)	215(27.4)	16(19.8)	0.141
BMI, kg/m^2^	31.7 ± 8.1	31.8 ± 8.3	30.4 ± 6.4	0.074
Obesity	431(49.7)	393(45.3)	38(46.9)	0.597
SpO2, %	96.7 ± 2.1	96.7 ± 2.1	97.1 ± 1.5	0.079
HR, bpm	85(73–99)	84(73–98)	93(78–108)	0.003**
SBP, mmHg	145(132–163)	147(133–164)	138(121–152)	< 0.001^***^
DBP, mmHg	59(51–65)	58(52–65)	55(48–60)	< 0.001^***^
Hemoglobin, g/dL	10.2 ± 2.0	10.2 ± 2.0	10.2 ± 2.1	0.832
SCr, mEq/L	1.5(1.1–2.3)	1.5(1.1–2.2)	2.0(1.3–2.9)	0.003^**^
WBC, 10^9^/L	10.1(7.5–13.6)	10.1(7.5–13.5)	11.0(7.7–15.6)	0.135
PLT, 10^9^/L	218(165–283)	220(166–284)	203 (142–276)	0.191
RDW, %	15.6(14.5–17.1)	15.5(14.4–16.9)	16.3(15.4–17.8)	< 0.001^***^
AG, mEq/L	15.0 ± 3.5	14.9 ± 3.4	16.2 ± 4.3	< 0.001^***^
Chloride, mEq/L	102.4 ± 6.2	102.3 ± 6.1	100.7 ± 6.4	0.032^*^
Glucose, mg/dL	151(118–198)	151(117–195)	156(125–224)	0.313
Sodium, mEq/L	137.9 ± 4.9	138.0 ± 4.9	136.7 ± 5.4	0.025^*^
Potassium, mEq/L	4.3 ± 0.72	4.3 ± 0.71	4.5 ± 0.79	0.053
Bicarbonate, mEq/L	25.8 ± 5.7	25.9 ± 5.7	25.0 ± 5.6	0.182
PTT,sec	32.2(27.8–39.2)	32.2(27.7–39.2)	31.3(28.2–39.3)	0.830
BUN, mg/dL				< 0.001^***^
< 20	157(18.1)	155(19.7)	2(2.5)	
≥ 20	710(81.9)	631(80.3)	79(97.5)	
SOFA score	4(3-7)	4(3-6)	6(5-10)	< 0.001^***^
SAPSII	38(30–46)	37(30–45)	48(42–57)	< 0.001^***^
GWTG – HF score	39(34–45)	39(34–44)	48(43–52)	< 0.001^***^
Hospital LOS	9.2(5.9–14.9)	9.0(5.9–14.6)	11.4(6.7–20.7)	0.008^**^
ICU LOS, days				< 0.001^***^
≤ 3	422(48.7)	401(51.0)	21(25.9)	
>3	445(51.3)	385(49.0)	60(74.1)	
**Comorbidities, n (%)**				
ACS	49(5.4)	42(5.3)	7(8.6)	0.289
HP	303(34.9)	276(35.1)	27(33.3)	0.749
Hyperlipidemia	338(40.0)	308(39.2)	30(37.0)	0.706
COPD	52(6.0)	49(6.2)	3(3.7)	0.361
Shock	67(7.7)	41(5.2)	26(32.1)	< 0.001^***^
Stroke	14(1.6)	13(1.7)	1(1.2)	0.776
Ventricular	76(8.8)	66(8.4)	10(12.3)	0.231
prior-MI	131(15.1)	118(15.0)	13(16.0)	0.804
CKD	361(41.6)	332(42.2)	29(35.8)	0.263
AF	413(49.7)	358(45.5)	55(67.9)	< 0.001^***^
AKI	263(30.3)	223(28.4)	40(49.4)	< 0.001^***^
**Treatment strategy, n (%)**				
ACEI/ARBs	495(57.1)	479(60.9)	16(19.8)	< 0.001^***^
Insulin	733(84.5)	675(85.9)	58(71.6)	0.001^**^
Anticoagulant drug	350(40.4)	327(41.6)	23(28.4)/	0.021^*^
β-blockers	734(84.7)	680(86.5)	54(66.7)	< 0.001^***^
Digoxin	144(16.6)	129(16.4)	15(18.5)	0.628
Aspirin	570(65.7)	523(66.5)	47(58.0)	0.124
Diuretic	797(91.9)	725(92.2)	72(88.9)	0.292
Assisted ventilation	376(43.4)	331(42.1)	45(55.6)	0.010^*^
CRRT	59(6.8)	42(5.3)	17(21.0)	< 0.001^***^

Note: Data were presented as mean ± standard deviation (SD) or median (interquartile range, IQR) for skewed variables or numbers (proportions) for categorical variables

Abbreviations: BMI, body mass index; SpO2, pulse oximetry-derived oxygen saturation; HR, heart rate; SBP, systolic blood pressure; DBP, diastolic blood pressure; SCr, serum creatinine; WBC, white blood cell; PLT, platelet count; RDW, red blood cell distribution width; BUN, blood urea nitrogen; AG, anion gap; PTT, partial thromboplastin time; SAPS II, Simplified Acute Physiology Score II; SOFA score, Sequential Organ Failure Assessment; GWTG-HF, Guidelines-Heart Failure; LOS, length of stay; ICU, intensive care unit; ACS, acute coronary syndrome; HP, hypertension; COPD, chronic obstructive pulmonary disease; prior-MI, prior myocardial infarction; CKD, chronic renal dysfunction; AF, atrial fibrillation; AKI, acute renal impairment; ACEI/ARBs, angiotensin-converting enzyme inhibitors (ACEI) or angiotensin receptor blockers; CRRT, continuous renal replacement therapy; * *P* < 0.05; ** *P* < 0.01;*** *P* < 0.001

### Development of nomogram

**Fig. 2:**
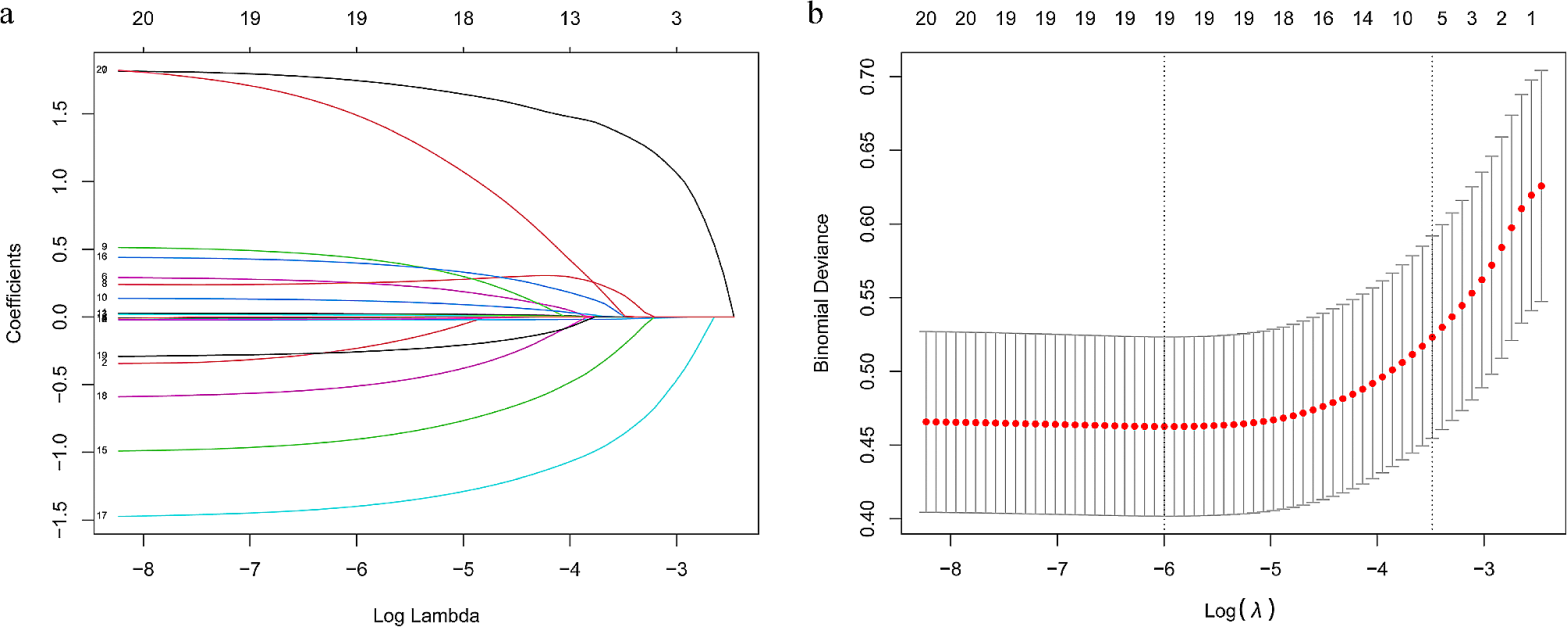
Clinical risk feature selection using the least absolute shrinkage and selection operator (LASSO) binary logistic regression model. (**a**) LASSO coefficient profiles of the 18 Clinical risk features. (**b**) Tuning parameter (λ) selection in the LASSO model used 10-fold cross-validation via minimum criteria

**Fig. 3:**
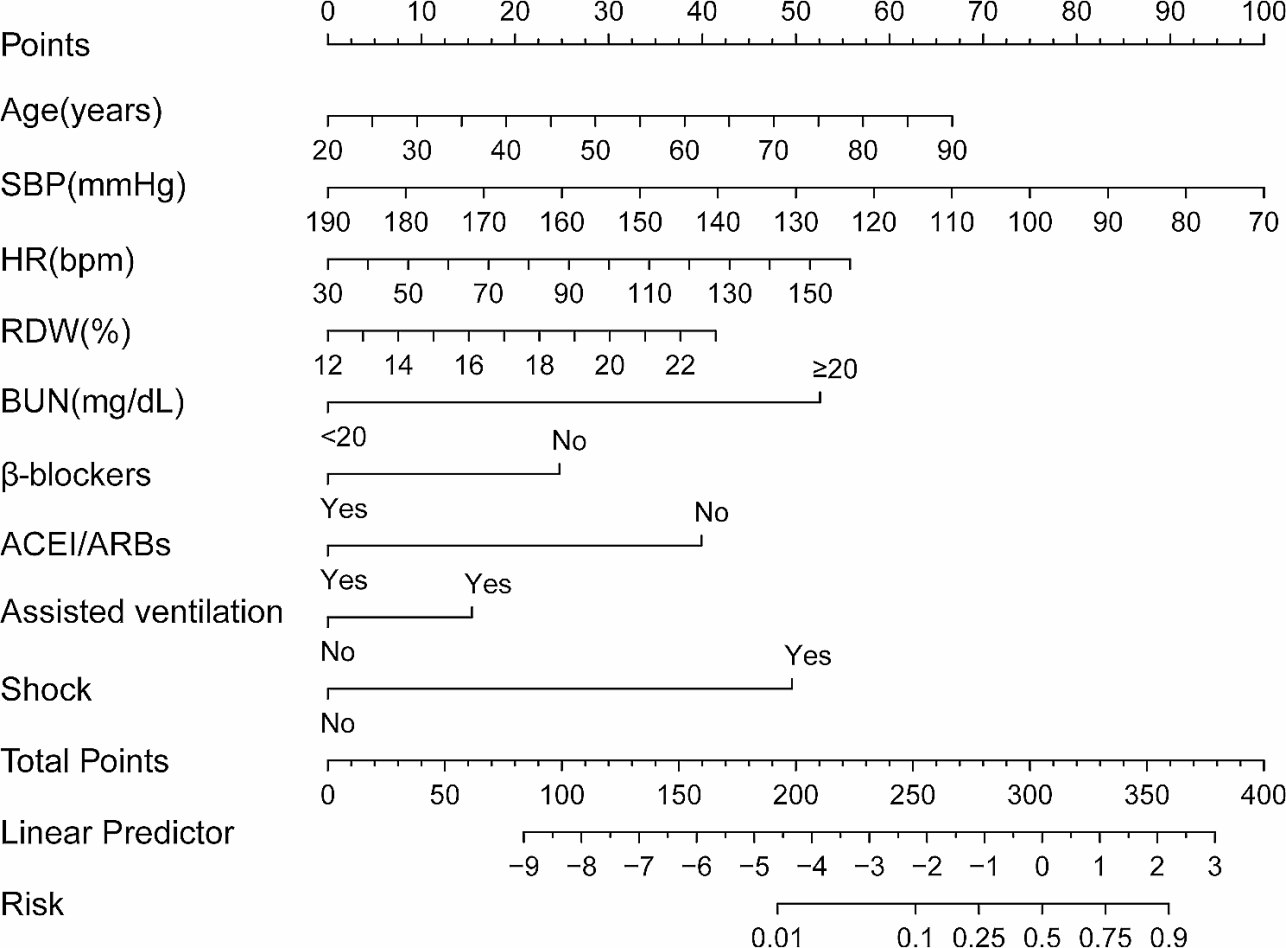
Nomogram to predict the risk of hospital death in patients with acute decompensated chronic heart failure and diabetes. RDW: Red blood cell Distribution Width; ACEI/ARBs: Angiotensin-Converting Enzyme Inhibitors /angiotensin receptor blockers; HR: Heart Rate; SBP: Systolic Blood Pressure; BUN: Blood Urea Nitrogen

Taking the occurrence of hospital death as the induced variable, the candidate variables with *P* < 0.05 in the Table 1 were put into the LASSO regression model, which was designed to avoid over-fitting by imposing penalties on the size of the model coefficients, and 17 variables with non-zero coefficients were selected (Fig. 2). Subsequently, we placed the screened variables into the logistics multiple regression model, and the results suggested that age, HR, RDW, SBP, shock, β-blockers, ACEI/ARBs, assisted ventilation, and BUN were independent factors to predict hospital mortality in ADHF and diabetes, use of β-blockers, SBP, and ACEI/ARBs were negatively correlated with hospital mortality (see Table 2). According to the above nine predictors, we established a nomogram model for hospital death in patients with ADCHF and diabetes (Fig. 3), a way to visualize the results of the logistic multiple regression model to facilitate rapid identification and treatment decision making by clinicians.

**Table 2:** Multi-factor Logistics regression analysis based on LASSO regression

Variables	β	OR (95%CI)	*P* value
Age, years	0.028	1.039(1.01–1.069)	0.007^**^
HR, bpm	0.019	1.019(1.003–1.005)	0.010^*^
SBP, mmHg	-0.023	0.977(0.956–0.999)	0.040^*^
DBP, mmHg	-0.022	0.978(0.943–1.015)	0.240
Shock (Yes)	1.910	6.756(3.119–14.635)	< 0.001^***^
AF (Yes)	-0.472	1.603(0.864–2.973)	0.134
AKI (Yes)	-0. 311	1.365(0.761–2.448)	0.296
CRRT	-0.260	1.297(0.559–3.009)	0.544
Assisted ventilation (Yes)	0.615	1.849(1.024–3.337)	0.041^*^
BUN ≥ 20 mg/dL	1.880	6.552(1.465–29.305)	0.014^*^
Chloride, mEq/L	-0.025	0.975(0.932–1.020)	0.274
AG, mEq/L	0.021	1.020(0.945–1.103)	0.601
RDW, %	0.142	1.153(1.004–1.323)	0.043^*^
ACEI/ARBs (Yes)	-1.503	0.222(0.116–0.482)	< 0.001^***^
Insulin (Yes)	0.587	0.556(0.278–1.115)	0.098
β-blockers (Yes)	-0.978	0.376(0.194–0.727)	0.004^**^
ICU LOS < 3 days	0.296	0.744(0.387–1.430)	0.375

Abbreviations: HR, heart rate; SBP, systolic blood pressure; DBP, diastolic blood pressure; RDW, red blood cell distribution width; BUN, blood urea nitrogen; AG, anion gap; AF, atrial fibrillation; AKI, acute renal impairment; ACEI/ARBs, angiotensin-converting enzyme inhibitors (ACEI) or angiotensin receptor blockers; CRRT, continuous renal replacement therapy; LOS, length of stay; ICU, intensive care unit; OR, odds ratio ^*^P < 0.05; ^**^P < 0.01; ^***^P < 0.001

### Evaluation and validation of nomogram

**Fig. 4:**
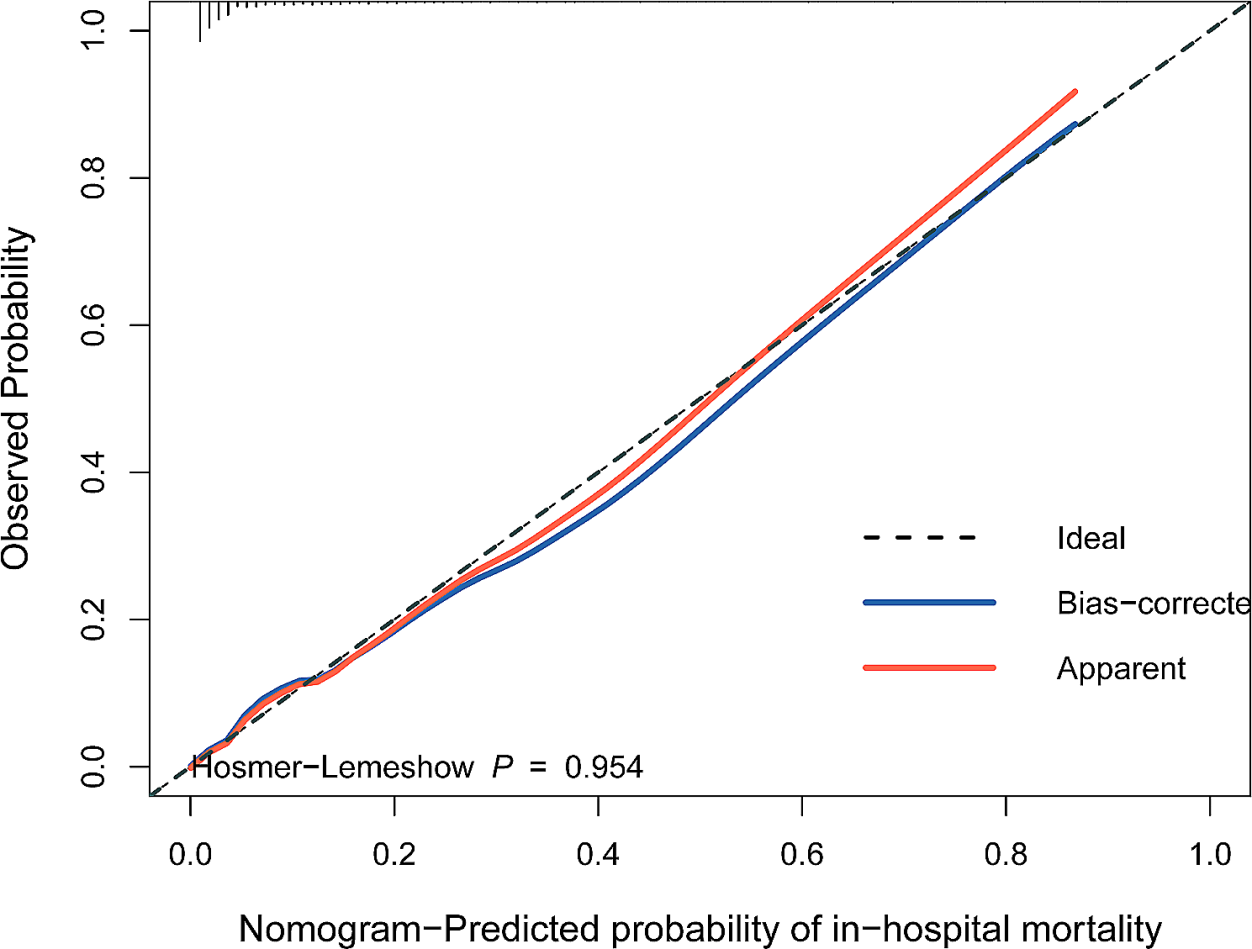
The Calibration curves of the nomogram model in patients with acute decompensated chronic heart failure and diabetes

**Fig. 5:**
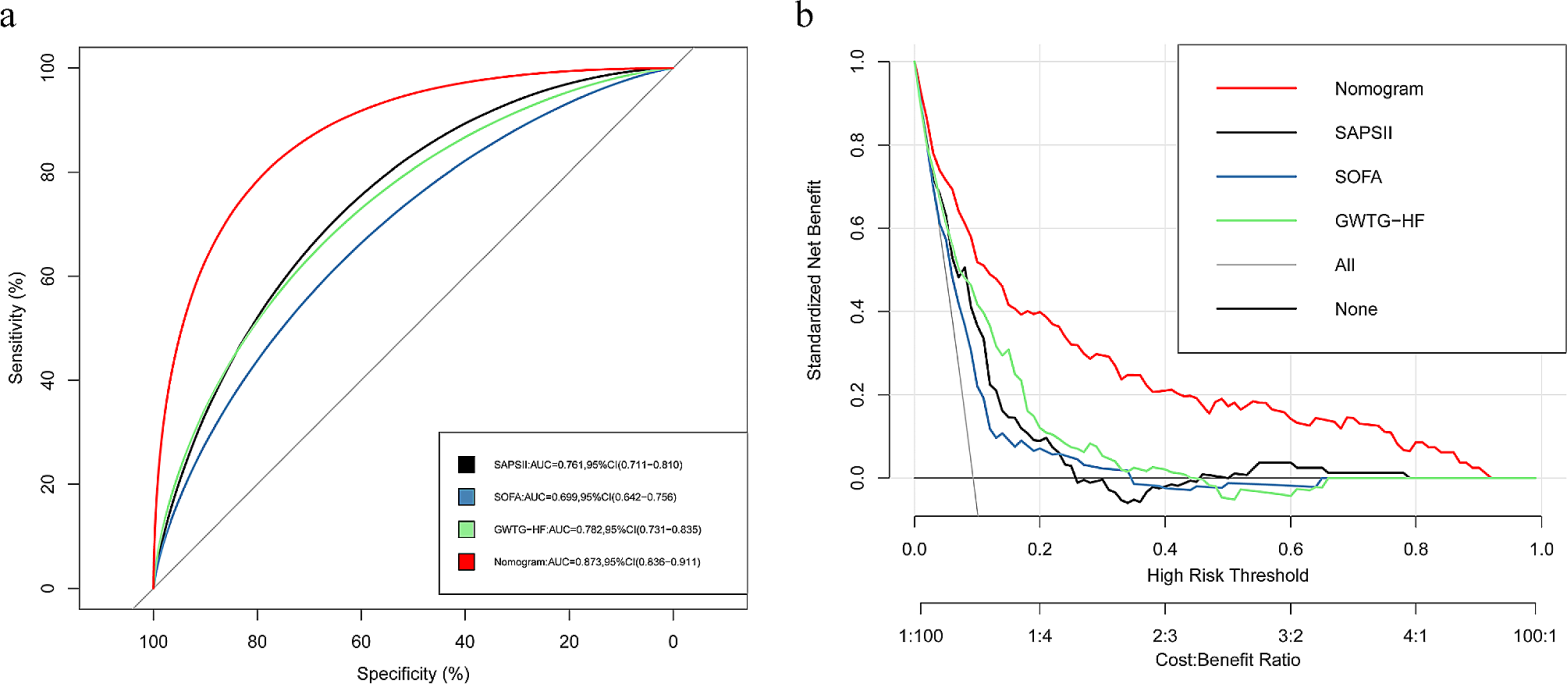
Receiver operating characteristics (ROC) curve analysis and decision curve analysis. (**a**) The ROC curve analysis and area under the ROC curve (AUC) (**b**) decision curve analysis. SAPS II: Simplified Acute Physiology Score; SOFA: Sequential Organ Failure Assessment; GWGT-HF: Guidelines-Heart Failure

At the same time, we also evaluated the performance of the nomogram, and the results were shown in Fig. 4, from which we could see that the red and blue curves were very close to the diagonal dotted line, and the H-L test result showed *P* = 0.954, suggesting that the nomogram had strong calibration capability. AUC, that is C-index, is an index that can be used to judge the distinguishing ability of various models. 0.6–0.75 is considered as a model with certain distinguishing ability, and > 0.75 is considered as a model with good distinguishing ability. As demonstrated in Fig. 5a, the AUC of the nomogram model was 0.873 (95%CI: 0.836–0.911), and that of SAPSII and SOFA score were 0.761 (95%CI: 0.711–0.810) and 0.699 (95%CI: 0.642–0.756), respectively. In addition, the Guidelines-Heart Failure (GWTG-HF) scoring system, which incorporates variables such as age, SBP, BUN, HR, serum sodium, and ethnicity, is a widely known and recognized system for scoring the risk of in-hospital mortality in patients with HF, and we calculated the GWTG-HF score for each patient, which was validated in this study, and the value of the AUC in this study population was 0.782 (95%CI: 0.731–0.835), suggesting that the prediction ability of the nomogram was obviously better than theirs. What’s more, the C-index of the nomogram model was 0.857 (95%CI: 0.825–0.891) by Bootstrap internal verification method. Furthermore, in order to further evaluate and verify the discrimination and predictive power of the model in different situations, we verified it in patients with shock vital signs, and the area under the ROC curve was conducted with an AUC of 0.876 (95% CI: 0.792–0.961), which suggested good discrimination (Supplementary Fig. 1). All in all, these results verified that the nomogram model of this study had good accuracy and discrimination.

In addition, we constructed a DCA to assess the clinical net benefit of the nomogram. When the predictive probability threshold was set at 0.1–0.95, the nomogram model curve (red) was above SAPSII (black), SOFA score (blue) as well as GWTG-HF (green), with relatively highest net benefit rates (Fig. 5b), suggesting that the nomogram was more informative for clinicians to develop individualized treatment and reduce hospital mortality.

## Discussion

At present, Nomogram is widely used in the medical field, which is more accurate and easier to understand in estimating the survival rate of patients with different diseases, and can better guide clinical decision-making. This study established and validated a new and well-performing predictive model that can be used to evaluate the risk of hospital death in patients with ADCHF and diabetes, especially in those patients after 48 hours of Intensive Care Unit. A nomogram was constructed based on nine key variables (age, HR, SBP, RDW, Shock, ACEI/ARBs, β-blockers, assisted ventilation, and BUN) screened by LASSO regression in this study. Compared with SAPSII, SOFA score, and GWTG-HF score, the nomogram model showed better calibration ability and clinical application value, especially the high C-index indicated that the nomogram model had good predictive ability.

Due to the large number of variables involved in this study, in order to avoid too many variables into the final model which might lead to overfitting and reduced clinical applicability, LASSO regression was adopted to select the key variables in this study, which was a linear regression that avoided overfitting by imposing penalties on the size of model coefficients, and selectively puts key variables into the model to obtain better performance parameters [12]. Therefore, we constructed the first visual nomogram that can calculate the hazard of hospital death in ADCHF and diabetes, and the C-index was 0.857, indicating that this model has good distinguishing ability. Importantly, the nine variables in this nomogram were available after admission and easy to calculate. It was convenient for clinicians to quickly assess the risk of hospital mortality after admission, identify high-risk patients, and provide early interventions to reduce hospital mortality, which had good clinical application value.

Of important, among the nine key variables screened by LASSO regression to plot the nomogram, Shock and age were more heavily weighted in the scores. Shock is the most serious manifestation of ADHF, accounting for approximately 5% of ADHF, and is an independent hazard factor for hospital mortality in patients with ADHF, with a mortality of 30–50% [13]. The occurrence of Shock in patients with ADCHF and diabetes was 7.7% (67/867), and hospital mortality reached 38.8% (26/67), with a strong association with high mortality, which was also reported by other studies [14]. Furthermore, since the patients of this study was ADCHF with diabetes, abnormal vascular endothelial function, abnormal myocardial electrophysiology and high thrombotic load caused by long-term blood glucose fluctuations make ADCHF more likely to progress to Shock, significantly increasing the risk of death [15]. Therefore, early diagnosis, early identification and early evaluation and treatment are very important to improve the clinical prognosis of patients as much as possible.

Advanced age is one important risk factor for poor prognosis in various cardiovascular diseases and is significantly correlated with sarcopenia [16], frailty [17] and multimorbidity, in addition to being a chronic inflammatory condition in itself. In our study, it was found that patients in the Death group were relatively older, which also confirmed that advanced age was related to a high risk of death. Appropriate nutritional intervention, physical activity and control of underlying diseases were particularly important. ACEI/ARBs and β-blockers have been repeatedly shown to reduce HF hospitalization rates and improve survival, and are the cornerstone of treatment for HF [18]. The imbalance of renin-angiotensin-aldosterone system (RAAS) is a characteristic of ADCHF combined with diabetes. Studies have shown that ACEI / ARBs can not only reduce the all-cause mortality and readmission rates of HF with diabetes, but also improve the renal function of patients by reducing proteinuria [19]. In recent years, several studies have reported that RDW is an inflammatory marker, favored by many researchers. It is associated with the severity and prognosis of various cardiovascular diseases including HF, and its potential mechanisms may be related to inflammation, oxidative stress, and ineffective erythropoiesis [20]. Xanthopoulos A et al. found that RDW was a marker of poor prognosis in AHF and DM patients among 218 AHF patients [21], consistent with the results of this study.

Moreover, assisted ventilation and BUN ≥ 20 mg/dL were included in the final model. Assisted ventilation, including non-invasive and mechanically assisted ventilation, is widely used in patients with ADHF, especially in those with acute pulmonary edema symptoms. to improve oxidation by reducing pulmonary edema, and is recommended as an effective treatment strategy [22], but its impact on mortality is unclear. Sharon, A et al. found that intermittent biphasic positive airway pressure was associated with increased acute myocardial infarction and in-hospital mortality in the treatment of ADHF [23]. Other researchers believe that assisted ventilation is an effective treatment strategy to improve the prognosis of ADHF [24]. In another real-world study, Yukino M, et al. found that the in-hospital mortality rate of 3927 patients with noninvasive ventilator was 5.9% higher than that of those without noninvasive ventilator (3.5%). Although there was no statistical difference, the trend was basically consistent with this study [25]. BUN is a sensitive indicator of hemodynamics and renal perfusion, and is a hazard for cardiovascular diseases such as ACS and ADHF [26, 27]. In a large registry of acute decompensated heart failure, the best single predictor of mortality among 39 potential clinical and laboratory variables was high BUN levels at admission [28]. Angraal S et al. developed a model using machine learning to predict readmission and all-cause mortality HF with preserved ejection fraction, in which BUN is one of the important predictors [29]. In a study on the epidemiology of hospitalized HF patients in China, zhang et al. found that high BUN levels were significantly associated with higher mortality, in addition to common variables such as infection, acute myocardial infarction, and low SBP as predictors of death [30]. In our study, similar result was found that BUN ≥ 20 mg/dL was an independent predictor of hospital death in patients with ADCHF and diabetes. Resting heart rate has previously been recognized a potential predictor of mortality in patients with chronic heart failure, but little is known about its role in patients with ADHF. One study found that HR at discharge was independently associated with 1-year mortality in patients with AHF [31]. In this study, in patients with ADCHF combined with diabetes, HR on admission was found to be independently associated with in-hospital mortality, which is consistent with the findings of Lancellotti, Patrizio et al [32].

A few limitations of this study were as following: Firstly, the study population was derived from a single-center ICU, which could not exclude selection bias and might limit the application of nomogram in a larger population and also required external validation of data from different health care institutions; Secondly, due to missing data values > 20%, N-terminal probrain natriuretic peptide (NT-proBNP) and left ventricular ejection fraction(LVEF), which were previously considered as independent risk factors, were not included in the model, and the model should be used with caution before evaluating these two conditions. Thirdly, the data of the study were extracted from the MIMIC-III database, which contains multiple years of data (2001–2012), during which the treatment of cardiovascular diseases, especially heart failure, has been continuously updated and may affect the application of the nomogram. Therefore, it is not yet fully confirmed whether it can be applied to the present population, and further confirmation of the nomogram is needed in the future for larger populations and depending on the year. Finally, because of the nature of retrospective studies, we might not be able to fully adjust for potential confounders, which would partially affect our results, but should not affect its validity.

## Conclusion

Overall, this study is groundbreaking in that we first developed and internally validated nomogram to predict hospital mortality risk for patients with ADCHF and diabetes after 48 hours in ICU. Clinicians can use this nomogram to easily and accurately assess the risk of patients, identify high-risk patients in time, and provide optimal individualized care and treatment. However, external validation is required, and further studies are also need to be carried out in order to validate whether individual interventions according to this nomogram will reduce hospital mortality in patients with ADCHF and diabetes.

### Electronic supplementary material

Below is the link to the electronic supplementary material.

Supplementary Material 1

Supplementary Material 2

## Data Availability

The datasets used and analyzed during the current study are available from the corresponding author on reasonable request.The website of MIMIC database: https://mimic.mit.edu/.

## Abbreviations

ADCHF: acute decompensated chronic heart failure
HF: heart failure
AHF: acute heart failure
MIMIC- III: Medical Information Mart for Intensive Care III
ICD: International Classification of Diseases
ICU: Intensive Care Unit
HR: heart rate
SBP: systolic blood pressure
DBP: diastolic blood pressure
RDW: red blood cell distribution width
Scr: serum creatinine
BUN: blood urea nitrogen
GA: glucose, anion gap
PTT: partial thromboplastin time
ACS: Acute coronary syndrome
COPD: Chronic obstructive pulmonary disease
HP: hypertension
prior-MI: prior myocardial infarction
CKD: chronic renal dysfunction
AF: atrial fibrillation
AKI: acute renal impairment
ACEI: angiotensin-converting enzyme inhibitors
ARBs: angiotensin receptor blockers
CRRT: continuous renal replacement therapy
SAPS II: Simplified Acute Physiology Score
SOFA: Sequential Organ Failure Assessment
GWGT-HF: Guidelines-Heart Failure
LASSO: least absolute shrinkage and selection operator
AUC: area under the receiver operating characteristic curve
DCA: decision curve analysis
C-index: consistency index
NT-proBNP: N-terminal probrain natriuretic peptide
LVEF: left ventricular ejection fraction
