# Supplementary material for: A new nomogram to predict in-hospital mortality in patients with acute decompensated chronic heart failure and diabetes after 48 Hours of Intensive Care Unit
Source: BMC Cardiovasc Disord. 2024 Apr 6;24:199. doi: 10.1186/s12872-024-03848-5 (PMC10998347; doi:10.1186/s12872-024-03848-5)
Supplement: Supplementary file 1 — Supplementary Material 1 [file 12872_2024_3848_MOESM1_ESM.docx]

| Supplementary Table 1 Details of missing values | | | | |
| --- | --- | --- | --- | --- |
| **Variables** | The number of Missing values | | The percent of Missing values | |
|  |  |  |  |  |
| Age | | 0 | | 0.0% |
| BMI | | 47 | | 5.4% |
| Height | | 47 | | 5.4% |
| Weight | | 23 | | 2.7% |
| SpO2 | | 20 | | 2.3% |
| HR | | 20 | | 2.3% |
| SBP | | 20 | | 2.3% |
| DBP | | 20 | | 2.3% |
| Hemoglobin | | 4 | | 0.5% |
| SCr | | 1 | | 0.1% |
| WBC | | 4 | | 0.5% |
| PLT | | 4 | | 0.5% |
| RDW | | 4 | | 0.5% |
| AG | | 1 | | 0.1% |
| Chloride | | 1 | | 0.1% |
| Glucose | | 7 | | 0.8% |
| Sodium | | 4 | | 0.5% |
| Potassium | | 4 | | 0.5% |
| Bicarbonate | | 43 | | 5.0% |
| PTT | | 26 | | 3.0% |
| BUN | | 1 | | 0.1% |
| NT-proBNP | | 708 | | 81.7% |
| HbA1c | | 662 | | 76.4% |
| Troponin | | 767 | | 88.5% |
| SOFA score | | 0 | | 0.0% |
| SAPSII | | 0 | | 0.0% |
| GWTG-HF score | | 20 | | 2.3% |
| Hospital LOS | | 0 | | 0.0% |
| ICU LOS | | 0 | | 0.0% |

Abbreviations: BMI, body mass index; SpO2, pulse oximetry-derived oxygen saturation; HR, heart rate; SBP, systolic blood pressure; DBP, diastolic blood pressure; SCr, serum creatinine; WBC, white blood cell; PLT, platelet count; RDW, red blood cell distribution width; BUN, blood urea nitrogen; AG, anion gap; PTT, partial thromboplastin time; SAPS II, Simplified Acute Physiology Score II; SOFA score, Sequential Organ Failure Assessment; GWTG-HF, Guidelines-Heart Failure; LOS, length of stay; ICU, intensive care unit.
