# Supplementary material for: A new nomogram to predict in-hospital mortality in patients with acute decompensated chronic heart failure and diabetes after 48 Hours of Intensive Care Unit
Source: BMC Cardiovasc Disord. 2024 Apr 6;24:199. doi: 10.1186/s12872-024-03848-5 (PMC10998347; doi:10.1186/s12872-024-03848-5)
Supplement: Supplementary file 2 — Supplementary Material 2 [file 12872_2024_3848_MOESM2_ESM.docx]

**Title: A new nomogram to predict in-hospital mortality in patients with acute decompensated chronic heart failure and diabetes after 48Hours of Intensive Care Unit**

**
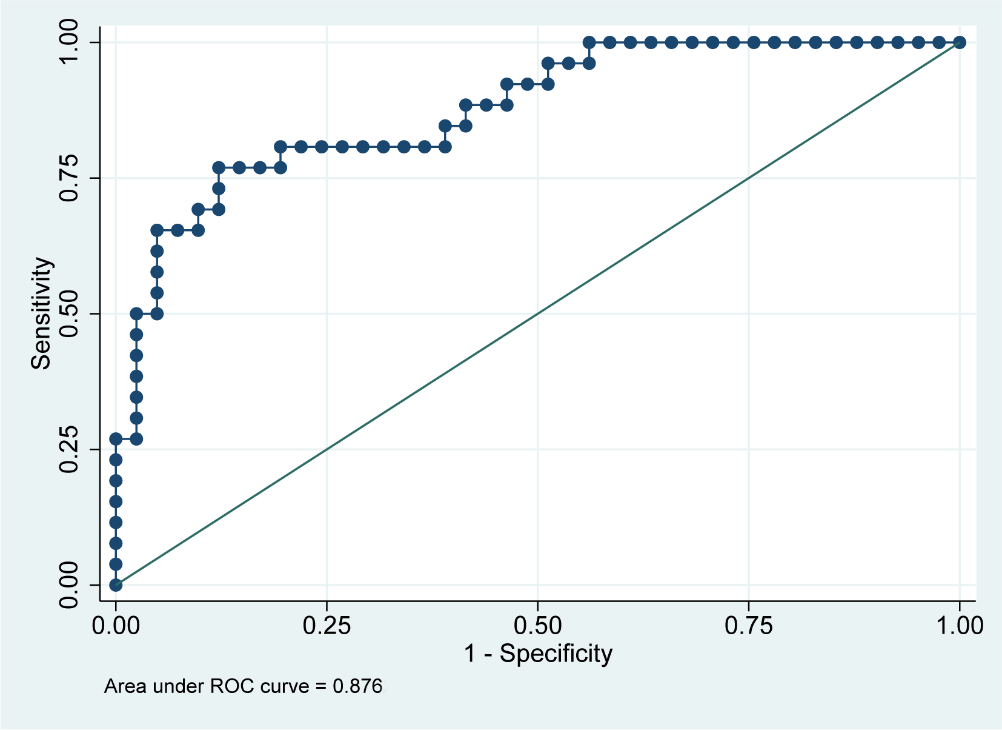
**

**Supplementary Figure 1** The ROC in patients among those who had shock vital signs. ROC: receiver operating characteristic; AUC: area under the curve.
